# O6-6 The added value of using the HEPA PAT for physical activity policy monitoring: A four-country comparison

**DOI:** 10.1093/eurpub/ckac094.046

**Published:** 2022-08-29

**Authors:** Peter Gelius, Sven Messing, Forberger Sarah, Jeroen Lakerveld, Mansergh Fiona, Taylor Sarah, Wanda Wendel-Vos, Joanna Zukowska, Catherine Woods

**Affiliations:** 1 Department of Sport Science and Sport, FAU Erlangen-Nürnberg, Erlangen, Germany; 2 Department Prevention and Evaluation, Leibniz-Institute for Prevention Research and Epidemiology - BIPS, Bremen, Germany; 3 Department of Epidemiology and Biostatistics, Amsterdam UMC, Amsterdam, The Netherlands; 4 Health and Wellbeing Programme, Department of Health, Dublin, Ireland; 5 Department of Physical Education and Sport Sciences, University of Limerick, Limerick, Ireland; 6 Physical Activity and Health Programme, National Institute for Public Health and the Environment RIVM, Bilthoven, The Netherlands; 7 Faculty of Civil and Environmental Engineering, Gdansk University of Technology, Gdansk, Poland

**Keywords:** physical activity recommendations, policy benchmarking, policy implementation, HEPA Policy Audit Tool, EU Monitoring Framework for HEPA across Sectors

## Abstract

**Background:**

Public policy is increasingly recognized as an important component of physical activity (PA) promotion, as policy actions to address lifestyle behaviours have the potential to in?uence the health and well-being of an entire population. However, our knowledge about the current status, implementation and effectiveness of PA policies in individual countries is still very limited, and there is consequently no clear guidance on which policies governments should preferably use in different settings or under various preconditions. In order to improve the evidence-base, we conducted a detailed assessment of existing PA policies in four EU Member States using WHO's HEPA Policy Audit Tool (PAT) in the context of the Policy Evaluation Network (PEN).

**Methods:**

We employed a six-step process to administer the HEPA PAT Version 2 in Ireland, the Netherlands, Germany, and Poland. This involved identifying stakeholders, pre-filling parts of the tool using existing survey data and desk-research, approaching select institutions to verify details, and obtaining expert opinion via workshops, interviews, and/or questionnaires. Based on the four completed PATs, we performed a comparative analysis to identify similarities and differences between countries and with previous studies using the tool.

**Results:**

In all four countries, the health and sport sector were found to be most active in PA promotion, followed by education, transport, and environment/urban planning. All countries have national systems to monitor population PA levels, and three out of four already have national PA recommendations. The study also showed that policy context (e.g. ministry portfolios, importance of subnational governments) varies substantially between countries. This influences policy implementation and made it necessary to employ a bespoke approach in each country to obtain the required information.

**Conclusions:**

Our findings largely confirm results of previous studies using the PAT in other countries. They also indicate that using the tool in combination with other policy monitoring tools, e.g. the EU Monitoring Framework for HEPA across Sectors, may provide added value and help countries monitor policy progress more consistently. Our experience also confirms some known limitations of the PAT, e.g. regarding subnational policies and a high level of dependence on cooperation from key policy actors.

